# Experimental and Thermodynamic Study on the Temperature-Dependent Surface Activity of Some Polyether Siloxane Surfactants at the Water–Air Interface

**DOI:** 10.3390/ijms26125472

**Published:** 2025-06-07

**Authors:** Joanna Krawczyk, Joanna Karasiewicz, Katarzyna Wojdat

**Affiliations:** 1 Department of Interfacial Phenomena, Institute of Chemical Sciences, Faculty of Chemistry, Maria Curie-Skłodowska University in Lublin, Maria Curie-Skłodowska Sq. 3, 20-031 Lublin, Poland; 2 Department of Chemistry and Technology of Silicon Compounds, Faculty of Chemistry, Adam Mickiewicz University in Poznań, Uniwersytetu Poznańskiego 8 Street, 61-614 Poznań, Poland

**Keywords:** trisiloxane surfactants, adsorption, thermodynamic parameters

## Abstract

Measurements of the surface tension of aqueous solutions of some trisiloxane surfactants containing various polyether groups (HOL7, HOL9, and HOL12) at 293 K, 303 K, and 313 K were performed. The studied surfactants were synthesized by hydrosilylation reaction and their structural analysis was carried out by the ^1^H NMR, ^13^C NMR, ^29^Si NMR, as well as FT-IR techniques. The thermal stability of HOL7, HOL9, and HOL12, as well as their molecular weight distributions, were also studied. On the basis of the obtained experimental results of the surface tension of aqueous solutions of HOL7, HOL9, and HOL12, the activity of the studied surfactants at the water–air interface was determined and discussed in the light of intermolecular interactions. Using the measured values of the surface tension, the Gibbs surface excess concentration, the area occupied by the surfactant molecule in the adsorption layer, and the standard Gibbs free energy of adsorption of the studied surfactants at the water–air interface were also calculated. Based on the obtained thermodynamic parameters of adsorption of the studied surfactants at the water–air interface, temperature, as well as a number of polyether groups in the hydrophilic part of surfactant, impact on particular surfactant adsorption was deduced. In general, the changes in the standard Gibbs free energy of adsorption of the studied surfactants at the water–air interface indicate that their adsorption tendency decreases with decreasing temperature. In addition, that tendency also diminishes as the number of the polyether groups in the hydrophilic part of the surfactant increases.

## 1. Introduction

Siloxane surfactants are compounds composed of a hydrophobic flexible permethylated siloxane backbone coupled with one or more hydrophilic groups, including a spacer (EO, PO, or a copolymer EO/PO units) and an end group (R). Functional groups can be attached by means of the Si–C or Si–O–C linkage. The most popular examples of silicone surfactants are those in which several poly(oxyalkylene) groups are combined with the polydimethylsiloxane backbone. This group of surfactants is used in a very wide range of practical applications in which classical (hydrocarbon) surfactants are ineffective. This fact is associated with their physicochemical properties. Particularly, they are surface active in aqueous and nonaqueous media. Moreover, they self-associate in the solution to form micelles, vesicles, liquid crystal phases, and microemulsions (with oils). Thus, their application includes plastic foam stabilization, as wetting and spreading agents and lubricants, as water-in-oil emulsifiers, and imparting silicone feeling to personal care products [1]. Especially, trisiloxane surfactants, often referred to as superspreaders or superwetters, are added to pesticides to enhance the activity and the rainfastness of the active substance by promoting rapid spreading over the hydrophobic surfaces of leaves. These surfactants are also added to other products, including cosmetics, polyurethane foams, textiles and fibers, paints and coatings, and car care products [1,2]. The Si–O–Si bond, which is relatively flexible, is responsible for the unique siloxane surfactant structure, enabling the adoption of various configurations and making the air–water interface densely packed by methyl groups. This structure of the surfactant adsorption layer permits the reduction of the water surface tension to about 20 mN/m, which is also closely related to the surface energy of –CH_3_ groups [1]. Similar to other nonionic surfactants, siloxane ones possess a low value of the critical micelle concentration [2]. The aggregation behavior of trisiloxane surfactants in the aqueous solutions has been studied in some detail. These properties of siloxane surfactants are similar to those of hydrocarbon ones. There are two significant differences. First, the trisiloxane hydrophobic group is much wider and shorter than, for example, a linear C_12_H_25_ group [2]. The length of the trisiloxane group is only about 9.7 Å compared with 15 Å for C_12_H_25_, while its volume is larger—530 Å^3^ compared with 350 Å^3^ for C_12_H_25_. This causes the type of aggregates formed by siloxane surfactants to be shifted toward a lower curvature. Secondly, the cloud point curve of nonionic trisiloxane surfactants is mostly governed by the size and number of the oxyethylene groups [2]. Few studies have been reported for ionic siloxane surfactants. Several anionic, cationic, and zwitterionic trisiloxane surfactants have been found to form micelles, vesicles, and lamellar liquid crystals. As expected, salt promotes a tendency toward smaller curvature structures [2,3]. The most effective and widely used method for polyether polysiloxane synthesis is the hydrosilylation process. A siloxane polymer, monomeric silane, or siloxane oligomer can be precursors with the Si–H bond as well as substituents susceptible to hydrolysis in the silicon atom vicinity. Platinum compounds are usually used in such reactions as catalysts [4,5,6,7]. The literature often responds to the application of the method to conduct the silicon polyether synthesis by means of the hydrothiolation reaction [8,9,10,11,12]. Good biodegradability, low toxicity, and the great multiplicity of the structure of the silicone surfactants are strictly related to their widespread use. Thus, siloxane surfactants have many applications in different fields—for example, in the textile and fiber industry [13,14], agriculture [15,16,17], personal care and cosmetics industry [2], and paints and coatings [18,19]. It should be mentioned that, despite a broad range of siloxane surfactant applications, the effect of temperature on the process of adsorption has not been fully studied [2,9,10,11,12,20,21,22,23]. Additionally, it is known that certain groups of silicone surfactants differ in terms of their stability and resistance to hydrolysis [2]. The stability of the molecule may also depend on the environmental conditions. One of the factors that may affect the stability of a silicone surfactant is temperature [2,16]. Liquid surface tension plays an important role in many processes. The two main factors influencing the magnitude of the liquid or solution surface tension are the chemical composition of the solution and temperature. Measurements of surface tension at different temperatures allow, among other things, the determination of basic thermodynamic functions that provide information about the direction and type of processes occurring in systems containing surfactants.

Moreover, one can find numerous discrepancies concerning the dependence of physicochemical properties of siloxane surfactants and their behavior in the processes of adsorption and micellization at various temperatures on the molecular structure of siloxane surfactants [2,3,16].

The objective of the research was to determine the adsorption properties of trisiloxane polyether surfactants such as 3-[3-(hydroxy)(polyethoxy)propyl]-1,1,1,3,5,5,5-heptamethyltrisiloxanes) (HOL7, HOL9, and HOL12) at various temperatures. They were examined at various concentrations of surfactants and at temperatures of 293 K, 303 K, and 313 K from the measurements of surface tension. The surfactant synthesis was performed as described earlier [19,20], being characterized based on analyses using ^1^H NMR, ^29^Si NMR, ^13^C NMR, FT-IR, GPC (gel permeation chromatography), and TG (thermogravimetric analysis).

## 2. Results and Discussion

### 2.1. Results of Synthesis

**Scheme 1:**
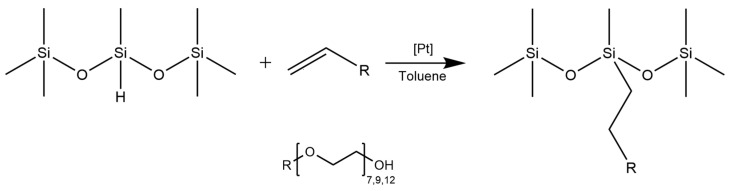
Hydrosilylation of allyl ether with 1,1,1,3,5,5,5-heptamethyltrisiloxane.

The synthesis of surfactants of siloxane polyether was conducted with allyl polyether including 7, 9, or 12 ethoxy (EO) groups, together with the terminal hydroxyl group. Thus, a compound characterized by amphiphilic properties was obtained. Toluene was used as a solvent in all reactions. The commercial Karstedt catalyst was also applied (Scheme 1).

The isolated product was subjected to spectroscopic analyses (^1^H NMR, ^13^C NMR, ^29^Si NMR) (Figures S1–S9). The results of the spectroscopic analysis of HOL7 and HOL12 have been presented previously [19,20]. The above-mentioned results for HOL9 are presented below:

Product: HOL9

- ^1^H NMR (CDCl_3_, TMS) δ (ppm): −0.05 (CDCl_3_, TMS) δ (ppm): −0.74 (–SiCH_3_); 1.49 (–Si(CH_3_)_3_); 13.11 (–SiCH_2_–); 22.79 (–CH_2_CH_2_CH_2_–); 61.19 (–CH_2_O–); 70.18 (–OCH_2_CH_2_–).
- ^1^Si NMR (CDCl_3_, TMS) δ (ppm): −21.76 (–Si(CH_3_)CH_2_); 6.98 (–Si(CH_3_)_3_).

**Figure 1:**
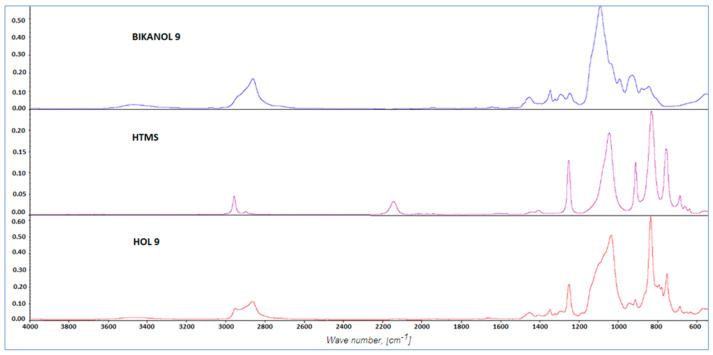
FT-IR spectra of the parent compounds and HOL9.

The representative substrate and product spectra for HOL9 are presented in Figure 1 where a band appears at ύ = 3500 cm^−1^, which can be due to the hydroxyl group stretching vibrations. The presence of this band in the product spectrum, and the disappearance of the bands at ύ = 2100 and 903 cm^−1^—due to the stretching vibrations of the Si–H group (present in the spectrum of the parent compound)—confirm the formation of the hydrosilylation product and not the occurrence of a condensation reaction between the Si–H and OH groups. The presence of bands related to symmetric and asymmetric stretching vibrations characteristic of methyl and methylene group C–H bands at ύ = 2700–3000 cm^−1^, as well as to the stretching vibrations of C–O–C bonds in the polyether chains and asymmetric stretching vibrations of Si–O–Si groups at ύ = 1000–1200 cm^−1^, confirms the structure of the product.

GPC was used for the determination of the molar mass distribution of the products (Figure S10). As follows from the data, the distribution of the products’ molecular weight was rather uniform. The surfactants were also investigated thermogravimetrically. Figures S11–S13 present the comparison of the TGA and DTG curves. Several stages of decomposition were observed in the polymer chains. Following the initial loss of solvents and adsorbed water in the sample, there was registered a step of decomposition with a complex shape around 473.15 K (HOL7 and HOL9) and around 523.15 K (HOL12), which was attributed to the cleavage of aliphatic fragments as well as the ether bonds in the functional group, consistent with the molecular structure. Decomposition of the siloxane backbone into dimethyl cyclosiloxane (DMC) occurred at about 773.15 K. The polymer decomposition temperature is determined based on the thermal stability of the surfactant molecules [2,20].

### 2.2. Tendency of Some Trisiloxane Polyether Surfactants to Adsorb at the Water–Air Interface at Various Temperatures

**Figure 2:**
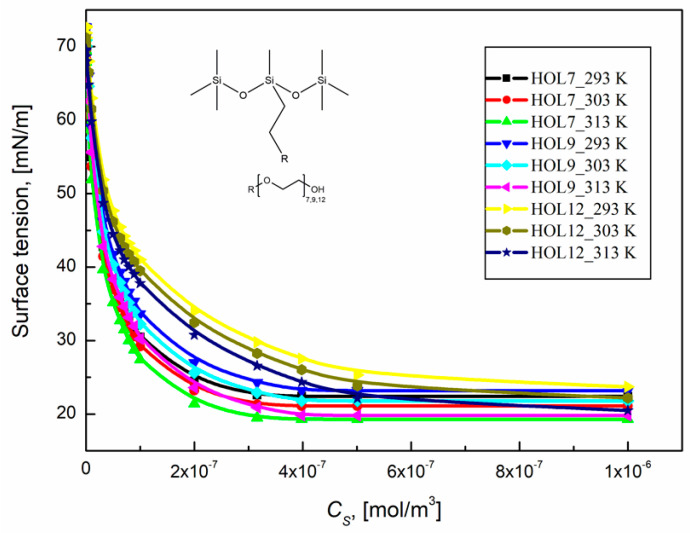
Plot of the surface tension ($\gamma_{LV}$) of aqueous solutions of HOL7, HOL9, and HOL12 at 298 K, 303 K, and 313 K vs. the surfactant concentration in the solution ($C_{S}$).

Changes in the values of the aqueous solution of the surfactant surface tension are due to its molecule adsorption process at the interface between water and air. This process depends largely on the polarity of the solute as well as the structural parameters of the surfactant molecule—that is, the structure of the hydrophilic and hydrophobic parts and their mutual proportion [20,21]. In the case of silicone surfactants, their adsorption properties depend on the length of the Si–O–Si backbone and the polymerization degree (number of oxyethylene (EO) groups) in the polar part. To determine the thermodynamic parameters of the adsorbed monolayer properties of trisiloxane surfactants (HOL7, HOL9, and HOL12) (Scheme 1) at the water–air interface, it is necessary to know the surface tension of the surfactants’ aqueous solutions at different temperatures. Figure 2 presents the values of the equilibrium surface tension ($\gamma_{LV}$) of trisiloxane polyether surfactants’ aqueous solutions at 293 K, 303 K, and 313 K. The obtained surface tension results for HOL12 are quite different from those presented earlier [19] because of some different surface tension measurement procedures as well as the instability of the labor solutions in time. To compare the surface activity of all studied surfactants (HOL7, HOL9, and HOL12), the conditions and procedure for the surface tension measurement must be the same. As follows from Figure 1 and literature data [2,3,20], in the case of the studied polyether trisiloxane surfactants, precise measurement conditions are crucial. Thus, in the paper, the surface tension of aqueous solutions of all studied surfactants was measured in the same way according to the procedure described below.

As can be seen in Figure 2, the values of aqueous solutions surface tension for the polyether trisiloxane surfactants are due to both temperature and the number of the EO units in the polar part of the surfactant molecule. Based on Figure 2, it can be stated that, similarly to other groups of surfactants (e.g., hydrocarbon), the surface tension of water decreases with increasing surfactant concentration ($C_{S}$) and temperature. However, comparing the obtained results for the trisiloxane surfactants at various temperatures with those in the literature is difficult, as data for 303 K and 313 K are not available. Moreover, for all studied trisiloxane polyether surfactants, in the range of concentration corresponding to the unsaturated monolayer at the interface between water and air, and over the entire temperature range, the relationship between $\gamma_{LV}$ and $C_{S}$ can be described using a second-order exponential equation (Figure 2). There is good agreement between the surface tension and some physicochemical parameter relations and the data previously presented for both other siloxane surfactants [24,25] and numerous classical hydrocarbon surfactants [26,27].

It is common that the minimal surface tension of the aqueous solution of a given surfactant depends particularly on the structure of the hydrophobic part of the surfactant molecule [21]. In the case of siloxane polyether surfactants, this structure is associated with the polymerization degree of the silicone backbone as well as the number of EO groups in the molecule. Based on the measured values of the surface tension of the aqueous solutions of the studied surfactants (HOL7, HOL9, and HOL12) (Figure 2), one can state that the minimal surface tension value depends slightly on the structure of the hydrophilic part of studied surfactants. This value increases to a small extent with the increasing hydrophilicity of a surfactant molecule of a given surfactant. In addition, in all cases, the minimal surface tension of aqueous solutions of the studied surfactants decreases with increasing temperature. As follows from the above, the surfactant molecules’ orientation at the interface between water and air changes with temperature, probably not being perpendicular, and differing from the *umbrella* type one [3]. Moreover, the chains of OE in the molecule of the siloxane surfactant are bent at the interface between water and air. This fact may affect the surfactant molecule area at the interface between water and air or the excess concentration of the surfactant. Taking into account the changes of the surface tension value with the surfactant concentration, the minimum surfactant concentration needed to form micelles (critical micelle concentration) (CMC) for the studied surfactants and under the studied conditions was determined from the breakpoint of the equilibrium surface tension vs. the surfactant concentration curve (Figure 2) (Table 1).

It was found that the *CMC* value for all studied surfactants decreases with increasing temperature. On the other hand, this value increases with the increasing degree of the polymerization of the EO group. Probably, this behavior is due to the changes in hydrophobicity of the surfactant molecule as well as the energy of the surfactant molecules, which increases with increasing temperature. To determine the adsorption properties of the studied surfactants, the values of $pC_{20}$ were also determined from the relations presented in Figure 2 at a given temperature ($pC_{20}$ corresponds to the negative logarithm of the molar concentration needed to decrease water (or other solvent) surface tension by 20 mN/m).

It is commonly known that the larger the $pC_{20}$ is, the more efficient a given surfactant adsorption is [21]. The above-mentioned parameter is strictly associated with the structural parameters influencing the adsorption and aggregation properties of the studied surfactants. The analysis of the obtained $pC_{20}$ and $\frac{CMC}{C_{20}}$ values shows the increasing siloxane surfactant tendency toward adsorption at the interface between water and air with increasing temperature and surfactant molecule hydrophobicity. Thus, it can be stated that HOL7 is characterized by the greatest tendency to adsorb at the water–air interface, compared to that to form micelles (at a given temperature). It is also worth noting that compared with conventional hydrocarbon surfactants, the large adsorption efficiency and effectiveness of the studied siloxane polyether surfactants’ adsorption are due to the flexibility of the Si–O–Si backbone, which leads to various configurations at the water–air interface. Moreover, the surfactants adsorb more easily at the interface between water and air than aggregate in the bulk phase due to steric inhibition of the siloxane surfactant molecule hydrophobic part [9]. According to the CMC values of the studied siloxane surfactants (Table 1), it can be also stated that increasing surfactant molecule hydrophobicity causes the *CMC* to increase. In addition, it is worth noticing that increasing temperature causes the *CMC* to decrease in the case of all studied trisiloxanes. The above may be due to the fact that the surfactants’ hydration degree decreases with increasing temperature.

The above results show good agreement with the earlier presented data [26,27,28]. Moreover, the obtained CMC values of the studied surfactants are in the range of those reported in the literature [3,8,9,10,12,29,30,31].

### 2.3. Activity of Surfactants at the Water–Air Interface at Different Temperatures

Based on the definition of chemical potential [32,33] of the *i* component of a solution, there is a relationship between the ratio of this potential in the surface layer to that in the bulk phase, the molar surface area, and the solution surface tension. However, the activity of a given component of a solution can be defined in two different ways: symmetrical (*a*) and asymmetrical (a*). Thus, *a* for a solvent and a solute approaches unity if x→1 (*x* is the molar fraction of a solvent or a solute), and a* = *x* for a solvent when x→1, but for a solute when x→0. In the case of the aqueous solution of surfactants, the above-mentioned dependence can be expressed by the Sprow and Prausnitz equation [26,34]:

(1) $$\gamma_{LV}=\gamma_{W}+\frac{RT}{\omega_{W}}\ln\frac{a_{W}^{S}}{a_{W}^{B}}$$

and

(2) $$\gamma_{LV}=\gamma_{S}+\frac{RT}{\omega_{S}}\ln\frac{a_{S}^{S}}{a_{S}^{B}}$$

or

(3) $$\gamma_{LV}=\gamma_{W}+\frac{RT}{\omega_{W}}\ln\frac{a_{W}^{*S}}{a_{W}^{*B}}$$

(4) $$\gamma_{LV}=\gamma_{S}+\frac{RT}{\omega_{S}}\ln\frac{a_{S}^{*S}}{a_{S}^{*B}}$$

where $\gamma_{LV}$ is the surface tension of the aqueous solution of surfactant, $\gamma_{W}$ is the surface tension of water, $\gamma_{S}$ is the surface tension of surfactants, $\omega_{W}$ is the molar area of water at the water–air interface, $\omega_{S}$ is the molar area of surfactant at the water–air interface, $a_{W}^{S}$ and $a_{W}^{B}$ or $a_{W}^{*S}$ and $a_{W}^{*B}$ are the activity of water in the surface layer and the bulk phase, and $a_{S}^{S}$ and $a_{S}^{B}$ or $a_{S}^{*S}$ and $a_{S}^{*B}$ are the activity of surfactant in the surface layer and the bulk phase, respectively.

Taking into account the fact that the surface tension of surfactant is different for the “tails” and “heads” [35], it is difficult to apply Equation (2) or Equation (4) to determine the surfactant activity in the surface layer based on its activity in the bulk phase. It is known that $\sum_{i=1}^{\alpha }a_{I}=1$ if a symmetrical definition of activity is considered, and in this case, the activity of surfactants can be determined using Equation (1).

In the case of studied surfactants, the adsorption process at the water–air interface was considered for the dilute solution of surfactants; thus, as a first approximation, it can be assumed that the activity of surfactant in the bulk phase is very small and that of water is close to unity. In such a case, Equation (1) can be expressed as follows [26]:

(5) $$\gamma_{LV}=\gamma_{W}+\frac{RT}{\omega_{W}}\ln a_{W}^{S}$$

Next, taking into account the molar area of water equal to 0.6023 × 10^5^ m^2^/mol [36], as well as the surface tension of laboratory solutions of surfactants (Figure 2) and that of water at a given temperature (72.8 mN/m at 293 K, 71.2 mN/m at 303 K, and 69.5 mN/m at 313 K), the activity of water in the surface layer was calculated from Equation (5). Based on the activity of water, that of the above-mentioned surfactants was calculated from the following relation: $a_{S}^{S}=1-a_{W}^{S}$.

**Figure 3:**
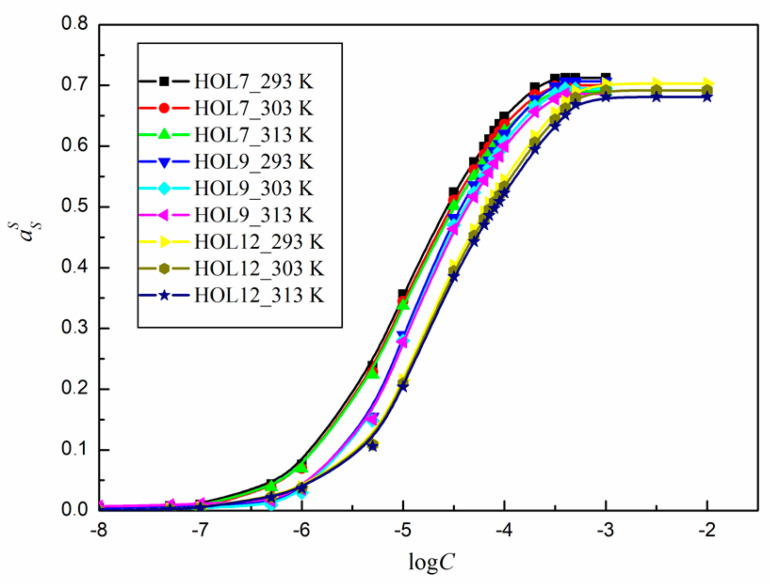
Plot of surface activity ($a_{S}^{S}$) of the aqueous solutions of HOL7, HOL9, and HOL12 at 298 K, 303 K, and 313 K vs. the logarithm of the surfactant concentration in the solution ($\log C_{S}$).

The obtained results are presented in Figure 3. The highest activity was observed for HOL7, with the smallest EO group polymerization degree, and the lowest for HOL12. It was also found that the $a_{S}^{S}$ values decrease with increasing temperature in the case of all studied trisiloxane surfactants.

**Figure 4:**
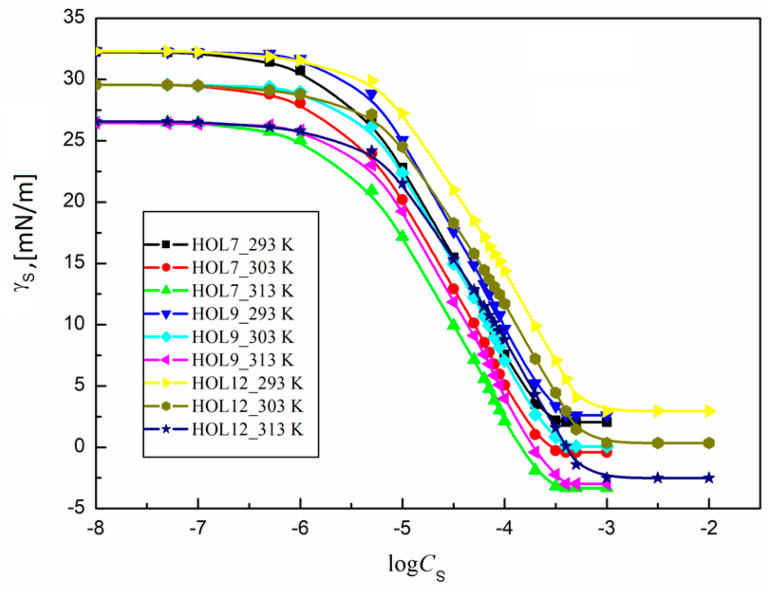
Dependence between HOL7, HOL9, and HOL12 surface tension, $\gamma_{S}$, determined based on Equation (6), and log*C* at various temperatures.

The activity of surfactants satisfies the following equation [26,37]:

(6) $$\pi =\pi_{\max}a_{A}^{S}$$

where *π* is the difference between $\gamma_{W}$ and $\gamma_{LV}$, and *π*_max_ is the difference between $\gamma_{W}$ and $\gamma_{S}$, respectively. If the activity of the studied trisiloxane surfactants, determined based on Equation (5), is reliable, $\gamma_{S}$ calculated from Equation (6) should be constant in the whole range of studied surfactants concentration. In the case of HOL7, HOL9, and HOL12, it appeared that $\gamma_{S}$ is constant only in the range of surfactant concentration (*C_S_*) corresponding to the almost linear dependence between $\gamma_{LV}$ and *C* and below the concentration at which a saturated monolayer at the interface between water and air is formed (Figure 4).

This relation is the same as in the case of some hydrophobic surfactants studied earlier [26]. In this range of *C*, the value of $\gamma_{S}$ is almost the same for HOL7, HOL9, and HOL12. At 293 K, it is equal to about 32.3 mN/m; at 303 K, it is equal to about 29.55 mN/m; and at 313 K, it is equal to about 26.52 mN/m (Figure 3). This suggests that in this range of surfactant concentration, its tail is oriented parallel to the water–air interface, and there are no interactions between the tails, and that the maximal decrease in water surface tension at such orientation of surfactant tails is equal to 72.8–32.3 mN/m (at 293 K) [26]. On the other hand, it is possible that in this range of *C*, the water molecules at the water–air interface are present in the same form. A decrease in $\gamma_{S}$ at *C* below that corresponding to the saturated monolayer of the adsorbed surfactant can suggest that the process of aggregation of surfactant molecules takes place at lower *C* than in the bulk phase and/or that $\omega_{W}$ increases [26]. It is worth emphasizing that there is practically no difference between the minimal $\gamma_{S}$ for all studied surfactants at low concentration.

### 2.4. Thermodynamic Parameters of the Trisiloxane Surfactants at the Interface Between Water and Air

The amount of studied siloxane polyether surfactants adsorbed per unit area at the air–water interface (the Gibbs surface excess concentration of surfactant at the water–air interface ($\Gamma_{S}$)), can be determined based on the equilibrium surface tension ($\gamma_{LV}$) data (Figure 2) and using the Gibbs adsorption equation [21,38,39].

This equation for dilute solutions (10^−2^ M/dm^3^ or less) of surfactants is as follows [21]:

(7) $$\Gamma_{S}=-\frac{C_{S}}{nRT}{\left(\frac{\partial \gamma_{LV}}{\partial C_{S}}\right)}_{T}=-\frac{1}{nRT}{\left(\frac{\partial \gamma_{LV}}{\partial \ln C_{S}}\right)}_{T}=-\frac{1}{2.303nRT}{\left(\frac{\partial \gamma_{LV}}{\partial \log C_{S}}\right)}_{T}$$

where *R* is the gas constant, *T* is the absolute temperature, $\gamma_{LV}$ is the surfactant solution surface tension, $C_{S}$ is the concentration of surfactant, and *n* is the constant which depends on the kind of surfactant (for nonionic surfactants it is equal to 1).

For the studied trisiloxane surfactants corresponding to the unsaturated adsorption monolayer at the water–air interface, $\Gamma_{S}$ was determined from Equation (7) and using the second-order polynomial equations describing the relationship between $\gamma_{LV}$ and $C_{S}$ of a given surfactant at a given temperature (Figure 2). The maximal $\Gamma_{S}$ values ($\Gamma_{S}^{\max}$) corresponding to the saturated adsorption monolayer of surfactant at the water–air interface were determined from the linear dependence between $\gamma_{LV}$ and $\log C_{S}$ in every case for a given temperature. The $\Gamma_{S}^{\max}$ values for HOL7, HOL9, and HOL12 at a given temperature were then used for the calculation of the minimal area ($A_{S}^{\min}$) of surfactant molecule at the water–air interface. The obtained results of $\Gamma_{S}^{\max}$ and $A_{S}^{\min}$ are presented in Table 2.

**Figure 5:**
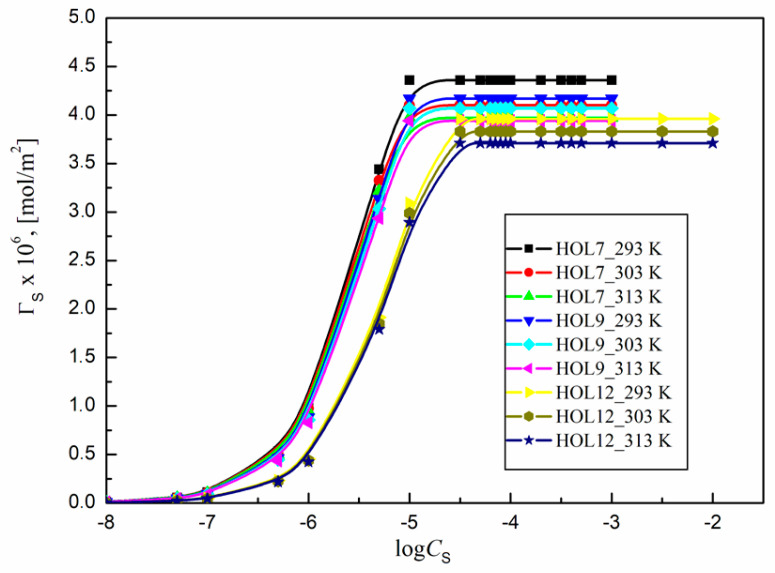
Plot of Gibbs surface excess concentration ($\Gamma_{S}$) of HOL7, HOL9, and HOL12 at 298 K, 303 K, and 313 K vs. the logarithm of surfactant concentration in the solution ($\log C_{S}$).

Both of these parameters describe the effectiveness of adsorption of the studied siloxane surfactants at the water–air interface [21]:

(8) $$A_{s}^{\min}=1/(N_{A}\Gamma_{S}^{\max)}$$

where $N_{A}$ is Avogadro’s number. Moreover, the changes in $\Gamma_{S}$ values for HOL7, HOL9, and HOL12 at 293 K, 303 K, and 313 K with the surfactant concentration in the bulk phase are presented in Figure 5. The $\Gamma_{S}^{\max}$ and $A_{S}^{\min}$ values of the studied trisiloxane surfactants at different temperatures are close to those reported in the literature [3,9,21]. However, a comparison of the obtained $\Gamma_{S}^{\max}$ values of the studied trisiloxane surfactants at 303 K and 313 K with those reported in the literature is difficult due to the lack of such data. As follows from the $\Gamma_{S}^{\max}$ data for the trisiloxane surfactants, the size of the polar part of the surfactant and temperature are decisive for their adsorption at the interface between water and air. Table 2 shows that the EO group polymerization increase causes the decrease in $\Gamma_{S}^{\max}$. A similar effect was observed in the case of temperature (Table 2). The temperature increase causes the $\Gamma_{S}^{\max}$ value to decrease for a given surfactant. This fact is probably associated with the flexibility of the silicone backbone of the studied siloxane surfactants as well as the OE chain in the polar part of the surfactant.

Based on the values of $\Gamma_{S}$ obtained from Equation (7) for HOL7, HOL9, and HOL12 at various temperatures (Figure 5), the mole fraction of the area occupied by the surfactant molecule at the interface between water and air ($X_{S}$) was determined from the following relation [21]:

(9) $$\Gamma_{W}N_{A}A_{W}^{0}+\sum \Gamma_{S}N_{A}A_{S}^{0}=1$$

where $\Gamma_{W}$ and $\Gamma_{S}$ are the surface excess concentrations of water and surfactant at the water–air interface, $A_{W}^{0}$ and $A_{S}^{0}$ are the limiting areas of water or surfactant molecule at the water–air interface, respectively, and

(10) $$X_{S}=\frac{\Gamma_{S}}{\sum \Gamma_{S}+\Gamma_{W}}$$

**Figure 6:**
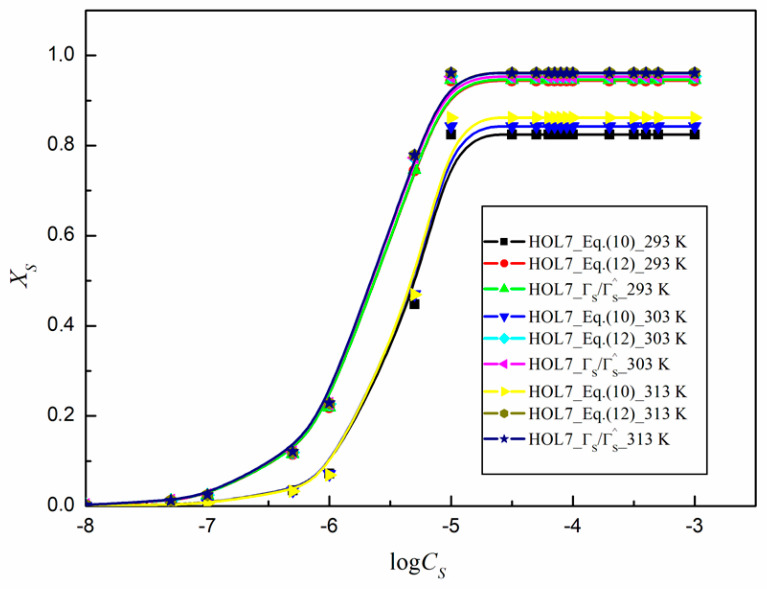
Plot of HOL7 mole fraction of the area occupied by the surfactant molecule in the adsorption layer ($X_{S}$) at the interface between water and air at 298 K, 303 K, and 313 K vs. the logarithm of surfactant concentration in the solution ($\log C_{S}$).

**Figure 7:**
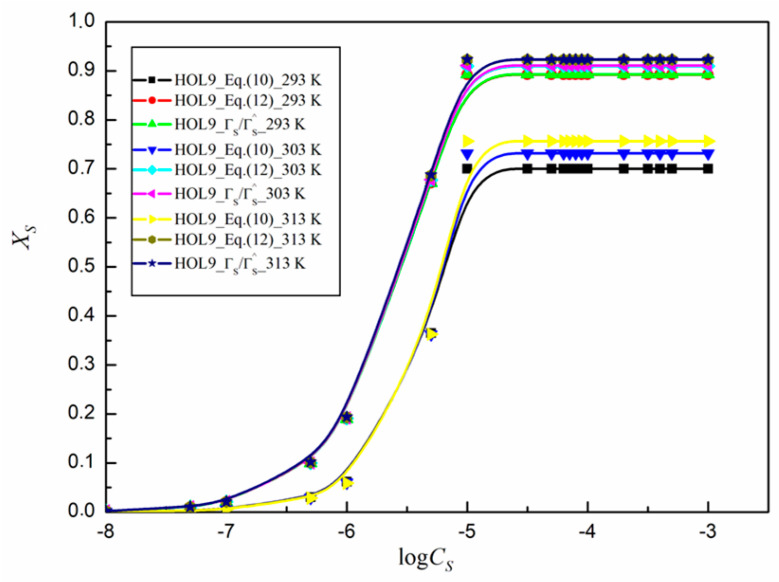
Plot of HOL9 mole fraction of the area occupied by the surfactant molecule in the adsorption layer ($X_{S}$) at the interface between water and air at 298 K, 303 K, and 313 K vs. the logarithm of surfactant concentration in the solution ($\log C_{S}$).

**Figure 8:**
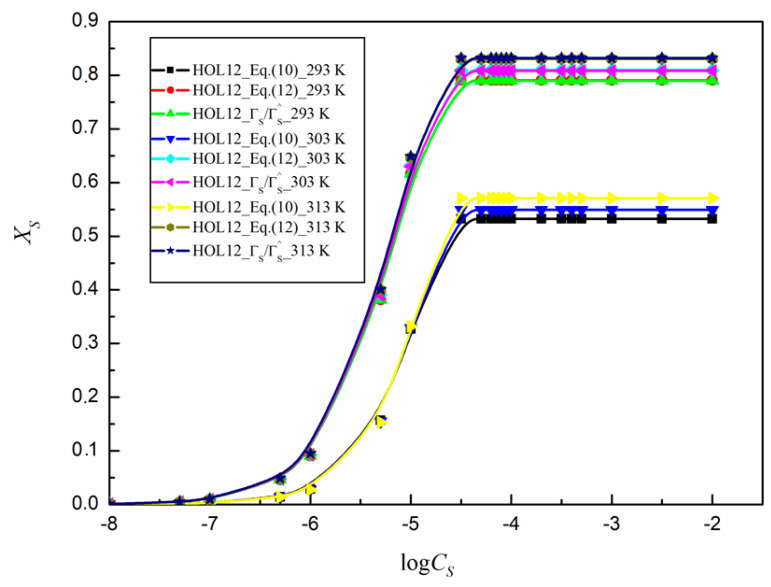
Plot of HOL12 mole fraction of the area occupied by the surfactant molecule in the adsorption layer ($X_{S}$) at the interface between water and air at 298 K, 303 K, and 313 K vs. the logarithm of surfactant concentration in the solution ($\log C_{S}$).

For calculations of $X_{S}$, a proper $A_{W}^{0}$ value at a given temperature was used. $A_{w}$ values at various temperatures were calculated by taking into account the increase of the distance between the water molecules in the surface region. On the other hand, the $A_{S}^{0}$ values for the studied surfactants can be determined from the Joos equation of state, which for the aqueous solutions of surfactants can be written in the following form [21]:

(11) $$\exp\left(\frac{-\pi }{RT\Gamma_{W}^{\infty }}\right)+\exp\left(\frac{-\pi }{RT\Gamma_{S}^{\infty }}\right)\frac{C}{a_{S}^{s}}=1$$

where $\Gamma_{W}^{\infty }$ and $\Gamma_{S}^{\infty }$ are the limiting Gibbs surface excess concentrations of water and surfactant at the interface between water and air, respectively, *π* is the film pressure, and $a_{S}^{s}$ is the activity of a studied surfactant at the interface between water and air. The $X_{S}$ values against $\log C_{S}$ for the studied polyether trisiloxane surfactants obtained from Equation (10) at different temperatures are presented in Figure 6, Figure 7 and Figure 8.

As follows from Figure 6, Figure 7 and Figure 8, the $X_{S}$ values calculated from Equation (10) for all studied polyether trisiloxane surfactants increase only slightly with temperature, but they decrease with the increasing degree of EO group polymerization in the hydrophilic part of the surfactant.

On the other hand, as it can be seen from Table 2, the $\Gamma_{S}^{\infty }$ values of the studied surfactant calculated from Equation (11) are somewhat higher than those of $\Gamma_{S}^{\max}$ in every case and temperature. Thus, based on $\frac{\Gamma_{S}}{\Gamma_{S}^{\infty }}$, it is possible to establish the extent of coverage at the interface between water and air by the surfactant molecules, and the obtained values should be equal to those determined from Equation (10) [39,40]. It was found that the values of $X_{S}$ for the particular trisiloxane surfactants obtained from the $\frac{\Gamma_{S}}{\Gamma_{S}^{\infty }}$ relation are significantly higher than those calculated from Equation (10), being close to 1. This indicates that the adsorption layer at the water–air interface is more densely packed than in the case of hydrophobic surfactants. This may result from the large flexibility of the studied surfactant molecules.

As it was stated in [39,40], the discrepancies may be due to different values of water and surfactant molecules’ limiting surface area. The number of water molecules that can be replaced by one surfactant molecule at the interface between water and air can be expressed as the ratio of $\frac{\Gamma_{S}^{\infty }}{\Gamma_{W}^{\infty }}$.

Assuming that $\frac{\Gamma_{S}^{\infty }}{\Gamma_{W}^{\infty }}=\frac{1}{k}$ [40], Equation (10) is as follows:

(12) $$X_{S}=\frac{1}{k}\frac{\Gamma_{S}}{\Gamma_{W}+\Gamma_{S}}$$

The values of $X_{S}$ for HOL7, HOL9, and HOL12 calculated from Equation (12) are practically equal to those obtained from the $\frac{\Gamma_{S}}{\Gamma_{S}^{\infty }}$ ratio (Figure 6, Figure 7 and Figure 8). Based on the $X_{S}$ values for HOL7, HOL9, and HOL12 and their changes with the temperature, it can be stated that the dehydration process of the polar head of the trisiloxane surfactant with different EO group degrees of polymerization probably proceeds to a smaller extent. To prove the above the analysis of the changes of the standard Gibbs free energy, enthalpy, and entropy of HOL7, HOL9, and HOL12 adsorption should be conducted.

### 2.5. Thermodynamics of the Adsorption of Some Trisiloxane Surfactants at the Water–Air Interface

Based on the relation between $\Gamma_{S}$ and $C_{S}$ for the studied trisiloxane surfactants at various temperatures (Figure 5), the standard Gibbs free energy ($\Delta G_{ads}^{0}$) of their adsorption at the water–air interface at a given temperature was determined.

In the case of dilute aqueous solutions whose concentration corresponds to the unsaturated monolayer at the water–air interface, the Langmuir equation can be used for the $\Delta G_{ads}^{0}$ determination [21,38,39,40]. Taking into account mutual interactions between the adsorbed molecules, the Langmuir equation assumes the following form [21,41]:

(13) $$\frac{A_{S}^{0}}{A_{S}-A_{S}^{0}}\exp\frac{A_{S}^{0}}{A_{S}-A_{S}^{0}}=\frac{C}{\omega }\exp\left(\frac{\Delta G_{ads}^{0}}{RT}\right)$$

where $A_{S}$ is the area occupied by the surfactant molecule at the interface, and ω is the number of water moles in 1 dm^3^ (at a given temperature).

**Figure 9:**
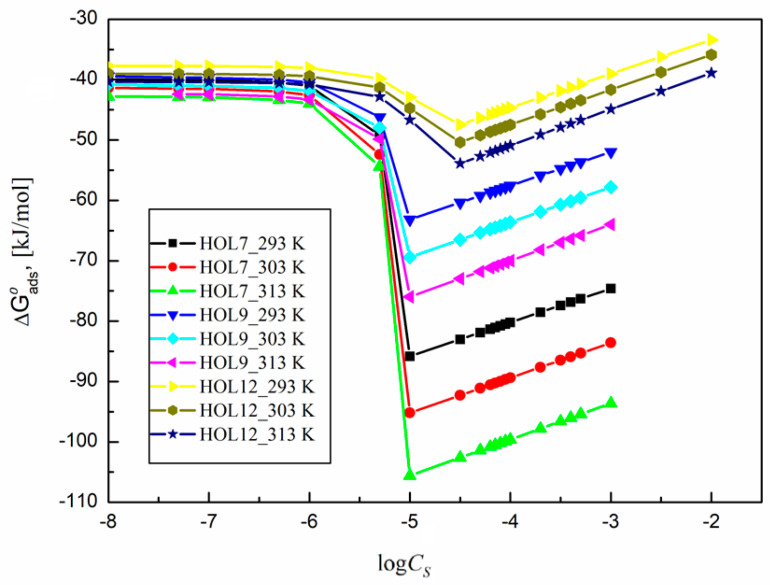
Plot of the standard Gibbs free energy of adsorption ($\Delta G_{ads}^{0}$) of HOL7, HOL9, and HOL12 at the water–air interface at 293, 303, and 313 K vs. the logarithm of its concentration ($\log C_{S}$).

To calculate $\Delta G_{ads}^{0}$ from Equation (13), the values of $A_{S}^{0}$ for a given surfactant must be known. $A_{S}$ values at the water–air interface for the studied surfactants at a given temperature are determined from the proper values of $\Gamma_{S}$ (Figure 5). The obtained $\Delta G_{ads}^{0}$ values of HOL7, HOL9, and HOL12 are presented in Figure 9. As follows from this figure, the $\Delta G_{ads}^{0}$ values calculated from Equation (13) are practically constant only in the range of surfactant concentration corresponding to the unsaturated monolayer at the interface between water and air (Figure 2). By analyzing the $\Delta G_{ads}^{0}$ values for individual surfactants in more detail, it was found that these values decrease (are more negative) with the increasing temperature and increase ($\Delta G_{ads}^{0}$ values are less negative) with the increasing degree of OE groups polymerization in the hydrophilic part of surfactant. The above effect is associated with changes in surfactant molecule hydrophobicity. There is good agreement between these results and the earlier data presented for silicone [42] and other surfactants [26,27,28,39,40].

According to the thermodynamic rule, $\Delta G_{ads}^{0}$ fulfills the following relationship [21,38]:

(14) $$\Delta G_{ads}^{o}=\Delta H_{ads}^{o}-T\Delta S_{ads}^{o}$$

$\Delta S_{ads}^{o}$ can be calculated from the following relation:

(15) $$\frac{d\Delta G_{ads}^{o}}{dT}=-\Delta S_{ads}^{o}$$

if $\Delta H_{ads}^{o}$ is constant over the investigated temperature range. To calculate the standard enthalpy of adsorption of the studied surfactant, the standard entropy of adsorption determined from the $\Delta G_{ads}^{0}$ (from Equation (13)), based on linear changes with temperature in the studied temperature range, was used. The obtained values of $\Delta H_{ads}^{o}$ and $\Delta S_{ads}^{o}$ for HOL7, HOL9, and HOL12 are presented in Table 3. The values of $\Delta S_{ads}^{o}$ and $\Delta H_{ads}^{o}$ are positive. Due to the fact that the values of $\Delta H_{ads}^{o}$ are affected by the hydrogen bonds formed between the molecules of water and hydrophobic surfactant molecules parts as well as surfactant molecules’ hydrophilic parts [21,38,39,40], the positive values of adsorption of entropy and enthalpy of HOL7, HOL9, and HOL12 indicate the predominance of bond breaking in the process of adsorption. In addition, the $\Delta H_{ads}^{o}$ values suggest that in the studied temperature range, dehydration of HOL7, HOL9, and HOL12 molecules takes place (Table 3).

## 3. Materials and Methods

### 3.1. Chemicals

All commercially available chemicals were used as purchased without any further purification. 1,1,1,3,5,5,5-heptamethyltrisiloxane was purchased from Sigma-Aldrich, St. Louis, MO, USA. Allyl polyethers (BIKANOL7, BIKANOL9, and BIKANOL12) were purchased from ICSO Chemical Production, Kędzierzyn-Koźle, Poland. The hydrosilylation catalyst was a commercially available Karstedt catalyst purchased from Sigma-Aldrich. For the preparation of the studied surfactant solutions HOL7, HOL9, and HOL12, doubly distilled and deionized water (Destamat Bi18E) (Heraeus, Hanau, Germany), which had an internal specific resistance of $18.2\times 10^{6}\;\Omega \cdot m$, was used. The purity of the water was additionally controlled by surface tension measurements before preparing the solutions.

### 3.2. Synthesis of 3-[3-(Hydroxy)(polyethoxy)propyl]-1,1,1,3,5,5,5-heptamethyltrisiloxane (HOL7, HOL9, and HOL12) Surfactants

The synthesis of the siloxane-containing polyether group using the hydrosilylation reaction of 1,1,1,3,5,5,5-heptamethyltrisiloxane and allyl polyether with 7, 9, or 12 ethoxy groups and a terminal hydroxy group (BIKANOL7, BIKANOL9, and BIKANOL12) was conducted. It proceeded in the presence of the Karstedt catalyst with toluene as a solvent. 1,1,1,3,5,5,5-heptamethyltrisiloxane (HMTS) was put into a three-necked round-bottom flask with a magnetic stirrer, a thermometer, and a reflux condenser. Next, it was dissolved in toluene. Then, after heating the mixture to 383.15 K (110 °C), the stechiometric amount of olefin was added. Next, there was added the amount of Karstedt catalyst corresponding to 1 × 10^−4^ mol Pt/1 mol of Si–H bonds. The temperature of the reaction mixture was kept at 383.15 K (110 °C) until the olefin substitution was complete. During the reaction, IR spectroscopy showed the band disappearance at 904 cm^−1^, which was assigned to the bond Si–H in the substrate. When the process was over, the post-reaction mixture was cooled, and the products were isolated by distilling off the excessive olefin and solvent under reduced pressure. The yields of the obtained pure product were high, being 97–99% (HOL7, HOL9, and HOL12). The assumed structure of the products was verified by spectroscopic analysis.

### 3.3. Physicochemical Studies

The nuclear magnetic resonance spectra: ^1^H NMR, ^13^C NMR, and ^29^Si NMR were obtained on a Bruker Ascend 400 (Bruker, Billica, MA, USA) at room temperature with CDCl3 as a solvent. The FT-IR spectra were collected using a Nicolet iS20 FT-IR spectrometer with a Gate diamond ATR attachment. The range of spectra collection was 500–4000 cm^−1^, and the resolution was 2 cm^−1^. Thirty-two scans of the background and sample were always recorded. Quantification of the reaction progress was based on observing the rate of band area change with the maximum at 904 cm^−1^, which could be assigned to the Si–H stretching vibrations.

GPC chromatograms were obtained using a Water Alliance 2695GPC system equipped with a Waters 2414 RI detector (Waters, Milford, MA, USA) and a set of three serially connected 7.8 mm × 300 mm columns (Waters Styragel HR1, HR2, and HR4). Molecular weights and polydispersity indices were calculated from the point-to-point calibration curve of polystyrene Shodex standards in the range of 1.32 × 10^3^ Da to 3.64 × 10^6^ Da. THF was used as an eluent at the isocratic flow rate of 0.6 mL/min.

TGA (thermogravimetric) analysis was performed using a Q50 apparatus (TA Instruments, New Castle, PA, USA), under nitrogen flow (60 mL/min) from room temperature to 1173.15 K (900 °C) at the heating rate of 283.15 K/min (10 °C/min).

### 3.4. Measurements of the Surface Tension

The measurement of water and the obtained aqueous solutions of HOL7, HOL9, and HOL12 for equilibrium surface tension was conducted at 293 K, 303 K, and 313 K using a Krüss KC100 tensiometer (Krüss, Hamburg, Germany) according to the platinum plate method. Before each measurement, the platinum plate was cleaned with acetone and then with distilled water and was heated to a red color with a Bunsen burner. The temperature was controlled by a jacketed vessel joined to the thermostatic water bath with an accuracy of ±0.01 K. The following surface tension measurement procedure was used: All the aqueous solutions at a given surfactant concentration lower than that at the stock solution were prepared just before the measurement by dilution from the proper stock solution. Next, the prepared solutions were mixed in the ultrasound bath for 15 min. After mixing, the laboratory solutions were immediately used for the measurement. At the beginning, the platinum plate was immersed into the solution. After 15 min, the measurement was started. Only one measurement for a given probe and at a given concentration was performed. In all cases, more than 10 successive measurements were performed. The standard deviation, depending on the surfactant concentration range, was from ±0.1 to ±0.2 mN/m.

## 4. Conclusions

The adsorption properties of some trisiloxane polyether surfactants (HOL7, HOL9, and HOL12) were studied in a wide range of surfactant concentrations at 293 K, 303 K, and 313 K based on the surface tension measurements. From the above-mentioned measurement results, the aggregation properties (*CMC*) of the studied surfactants were also determined. The analyses and interpretations of the obtained results allow us to draw the following conclusions: Similar to the other groups of surfactants, for all studied trisiloxane polyether surfactants, when the concentration range corresponds to the unsaturated monolayer at the interface between water and air over the whole range of temperature, it is possible to describe the $\gamma_{LV}$ and $C_{S}$ relation using a second-order exponential equation. Additionally, the value of the minimal surface tension increases to a small extent with the hydrophilicity increase of a molecule of a given surfactant. It was also found that for all studied surfactants, the minimal surface tension of their aqueous solutions decreases with increasing temperature. This fact suggests that the orientation of the trisiloxane surfactants molecules at the water–air interface can change with temperature.

## Figures and Tables

**Table 1:** The values of *CMC* and $pC_{20}$ (at 293 K, 303 K, and 313 K) of HOL7, HOL9, and HOL12.

Surfactant	*CMC* [mM/dm^3^]		
	293 K	303 K	313 K
HOL7	0.170	0.168	0.157
HOL9	0.200	0.198	0.195
HOL12	0.312	0.253	0.208

**Table 2:** The values of maximal Gibbs surface excess concentration ($\Gamma_{S}^{\max}$) and minimal area of HOL7, HOL9, and HOL12 molecules at the water–air interface ($A_{S}^{\min}$) at 293 K, 303 K, and 313 K.

Surfactant	$\Gamma_{S}^{\max}$ ×10^6^ [mol/m^2^]			$A_{S}^{\min}$ [Ǻ^2^]			$\Gamma_{S}^{\infty }$ ×10^6^ [mol/m^2^]			$A_{S}^{0}$ [Ǻ^2^]		
	293 K	303 K	313 K	293 K	303 K	313 K	293 K	303 K	313 K	293 K	303 K	313 K
HOL7	4.36	4.10	3.97	38.08	40.50	41.82	4.61	4.30	4.13	35.95	38.50	40.00
HOL9	4.17	4.07	3.94	39.82	40.72	42.06	4.67	4.47	4.27	35.53	37.00	38.70
HOL12	3.96	3.83	3.71	41.92	43.35	44.78	5.01	4.74	4.46	33.14	35.00	37.00

**Table 3:** The values of the standard Gibbs free energy ($\Delta G_{ads}^{0}$), enthalpy ($\Delta H_{ads}^{o}$), and entropy ($\Delta S_{ads}^{o}$) of HOL7, HOL9, and HOL12 adsorption at the interface between water and air at 293, 303, and 313 K.

Surfactant	$-\Delta G_{ads}^{0}$ [kJ/mol]		$\Delta H_{ads}^{o}$ [kJ/mol]	$\Delta S_{ads}^{o}$ [kJ/molK]
**HOL7**	**293 K**	39.990	0.141	1.323
	**303 K**	41.400	0.141	1.323
	**313 K**	42.810	0.141	1.323
**HOL9**	**293 K**	39.430	0.139	1.300
	**303 K**	40.810	0.139	1.300
	**313 K**	42.210	0.139	1.300
**HOL12**	**293 K**	37.960	0.130	1.262
	**303 K**	39.300	0.130	1.262
	**313 K**	40.640	0.130	1.262

## Data Availability

The original contributions presented in this study are included in the article/Supplementary Materials. Further inquiries can be directed to the corresponding authors.

## Supplementary Materials

The supporting information can be downloaded at: https://www.mdpi.com/article/10.3390/ijms26125472/s1.
