# Supplementary material for: Experimental and Thermodynamic Study on the Temperature-Dependent Surface Activity of Some Polyether Siloxane Surfactants at the Water–Air Interface
Source: Int J Mol Sci. 2025 Jun 7;26(12):5472. doi: 10.3390/ijms26125472 (PMC12192566; doi:10.3390/ijms26125472)
Supplement: Supplementary file 1 [file ijms-26-05472-s001.zip › ijms-3592521-supplementary.pdf]

Supplementary Materials

Experimental and Thermodynamic Study on the Temperature-Dependent Surface Activity of Some Polyether Siloxane Surfactants at the Water–Air Interface

Joanna Krawczyk [1,*], Joanna Karasiewicz [2,*] and Katarzyna Wojdat [1]

[1] Department of Interfacial Phenomena, Institute of Chemical Sciences, Faculty of Chemistry, Maria Curie-Skłodowska University in Lublin, Maria Curie-Skłodowska Sq. 3, 20-031 Lublin, Poland

[2] Department of Chemistry and Technology of Silicon Compounds, Faculty of Chemistry, Adam Mickiewicz University in Poznań, Uniwersytetu Poznańskiego 8 Street, 61-614 Poznań, Poland

* Correspondence: j.krawczyk@poczta.umcs.lublin.pl (J.K.); joanka@amu.edu.pl (J.K.); Tel.: +48-(81)-537-56-03 (Joanna Krawczyk); Fax: +48-(81)-533-33-48 (Joanna Krawczyk)

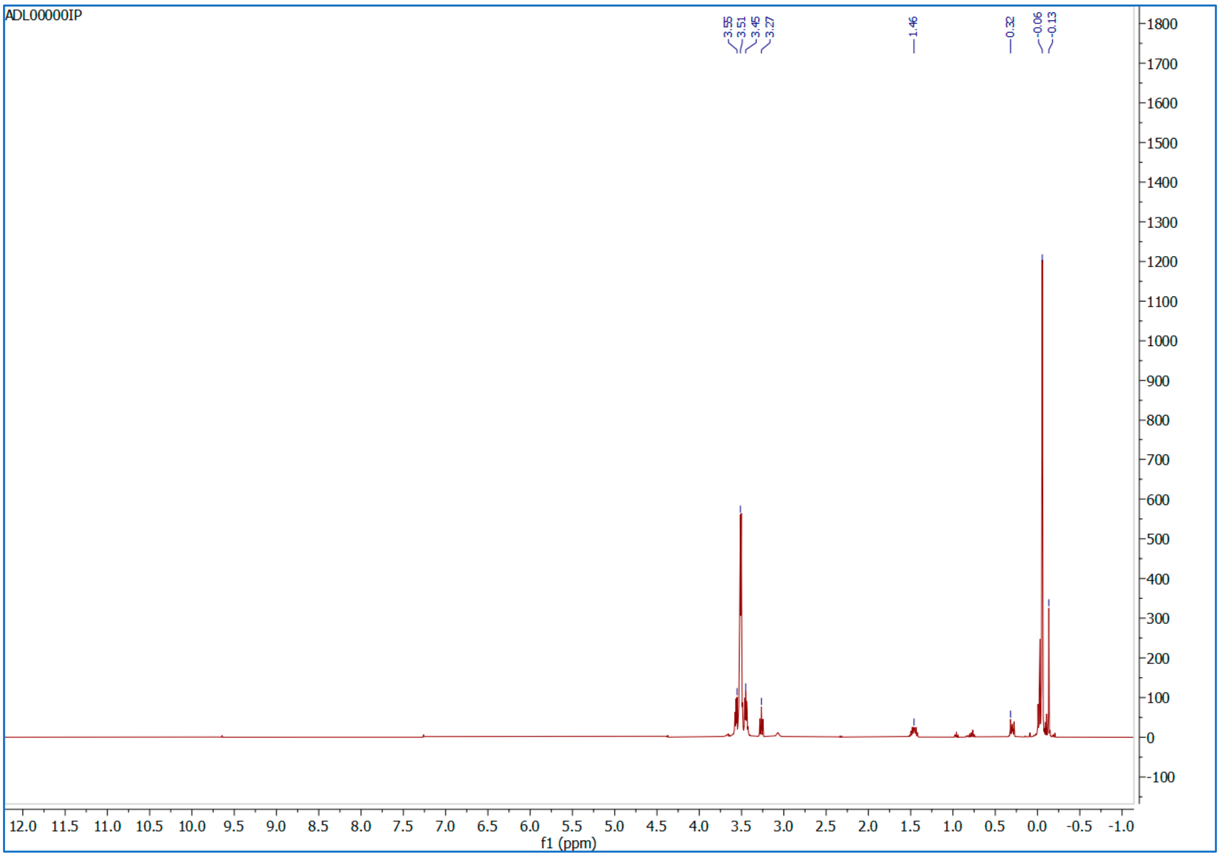

Figure S1. ^{1}H-NMR spectrum of HOL7.

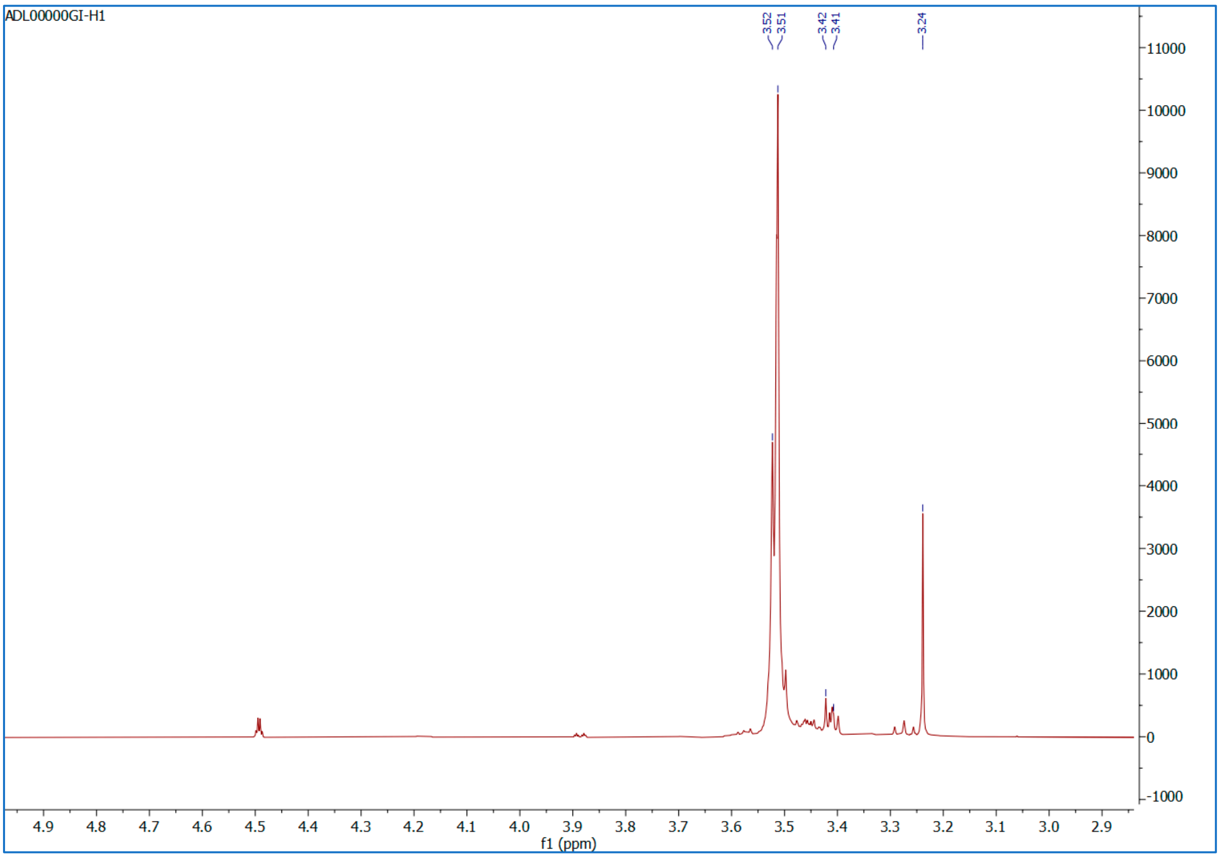

Figure S2. ^{1}H-NMR spectrum of HOL9.

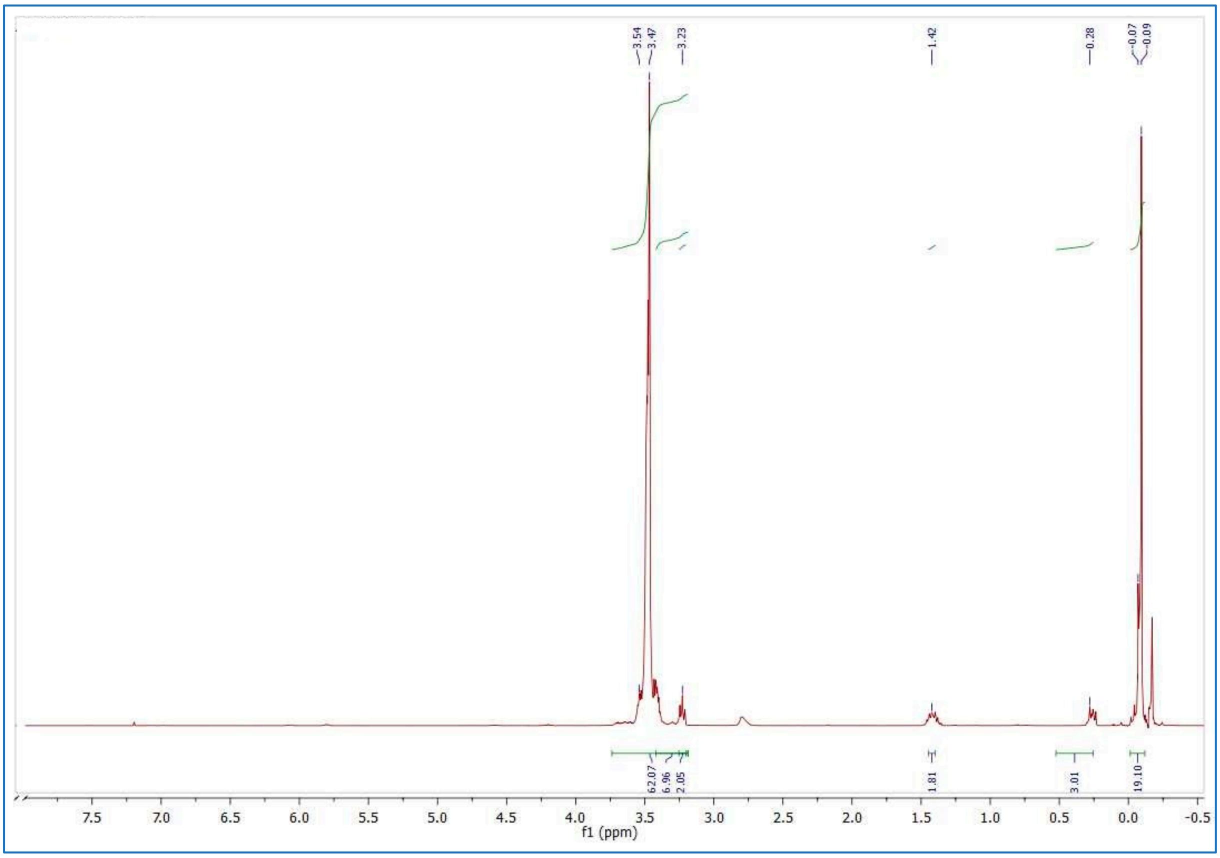

Figure S3. ^{1}H-NMR spectrum of HOL12.

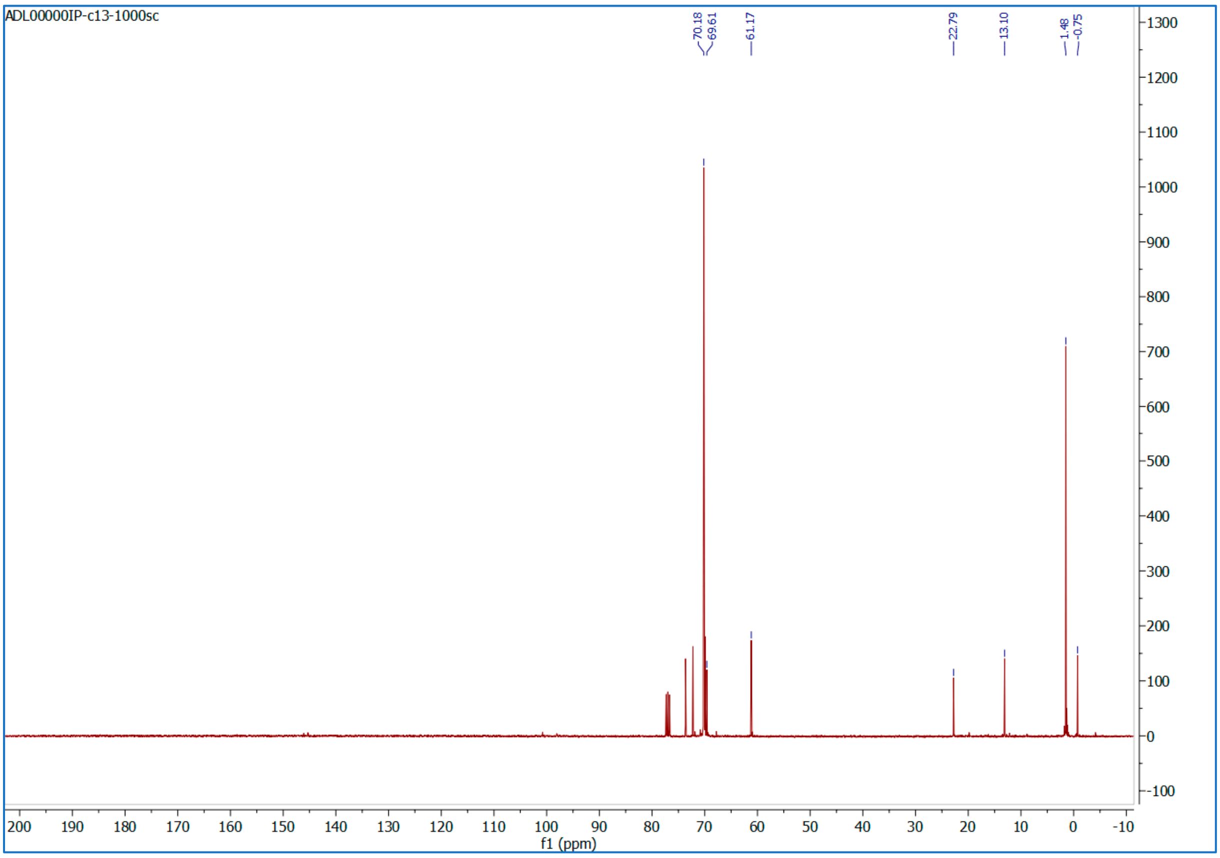

Figure S4. ^{13}C-NMR spectrum of HOL7.

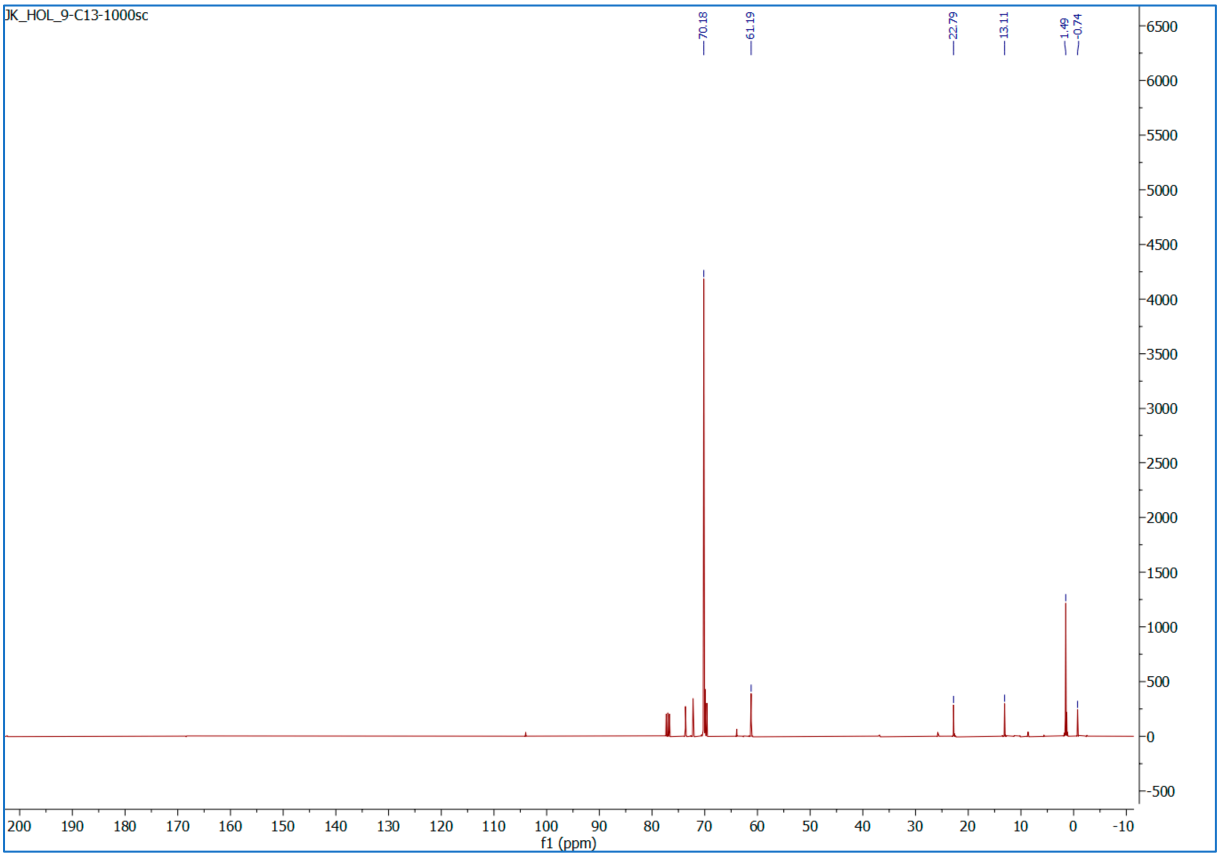

Figure S5. ^{13}C-NMR spectrum of HOL9.

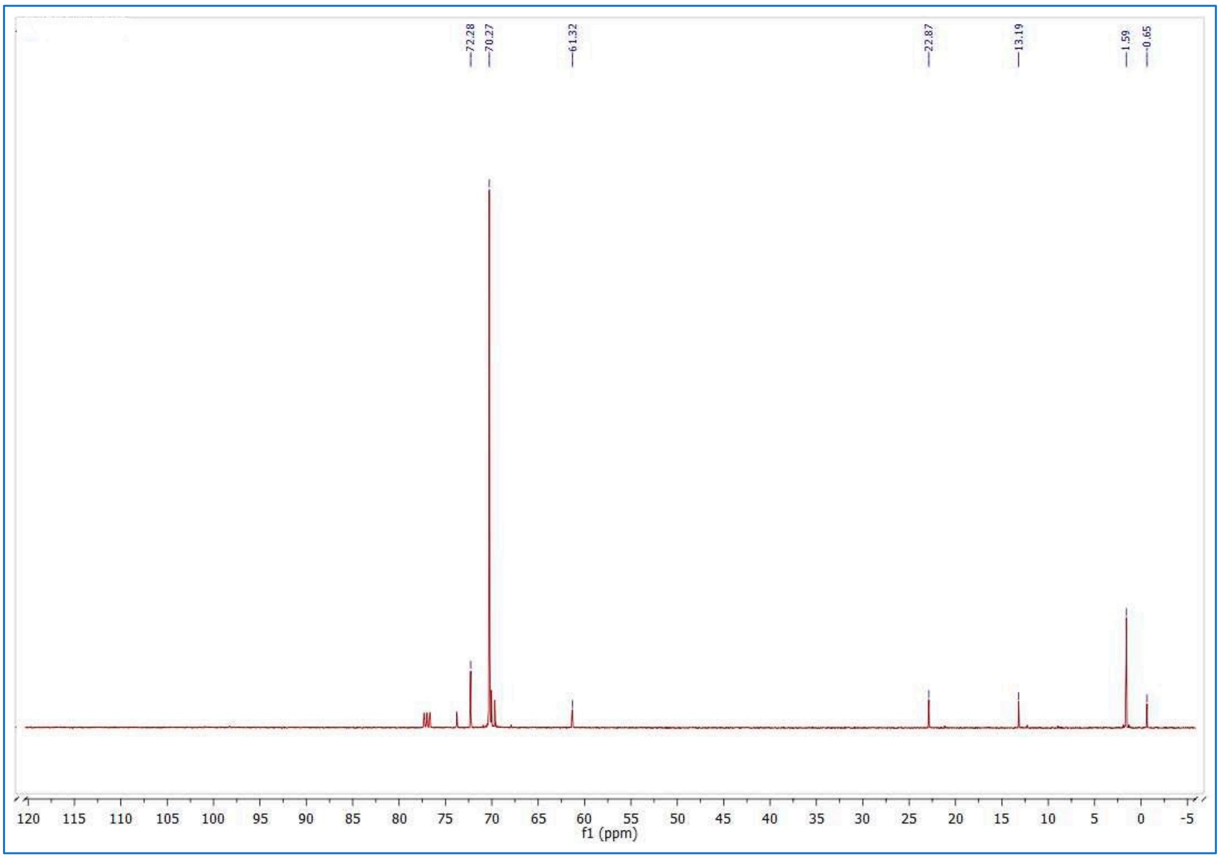

Figure S6. ^{13}C-NMR spectrum of HOL12.

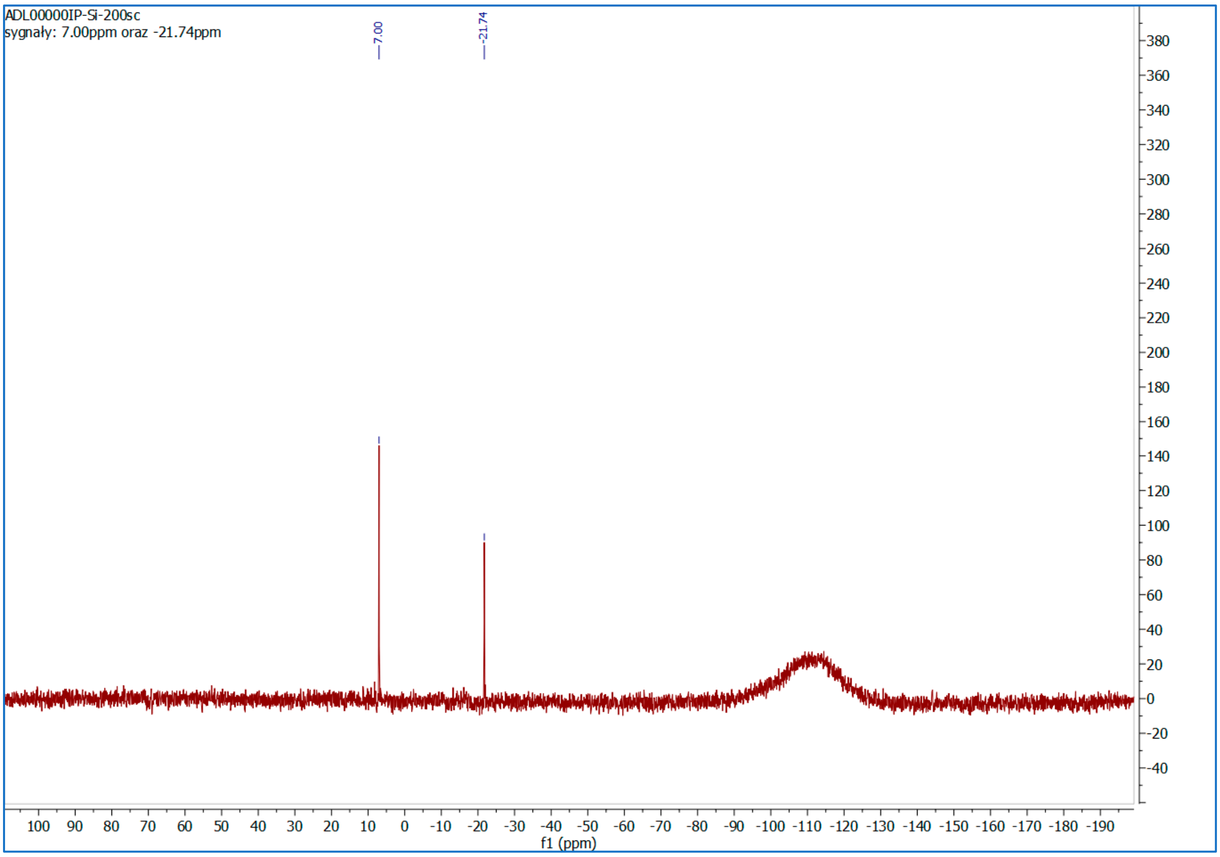

Figure S7. ^{29}Si-NMR spectrum of HOL7.

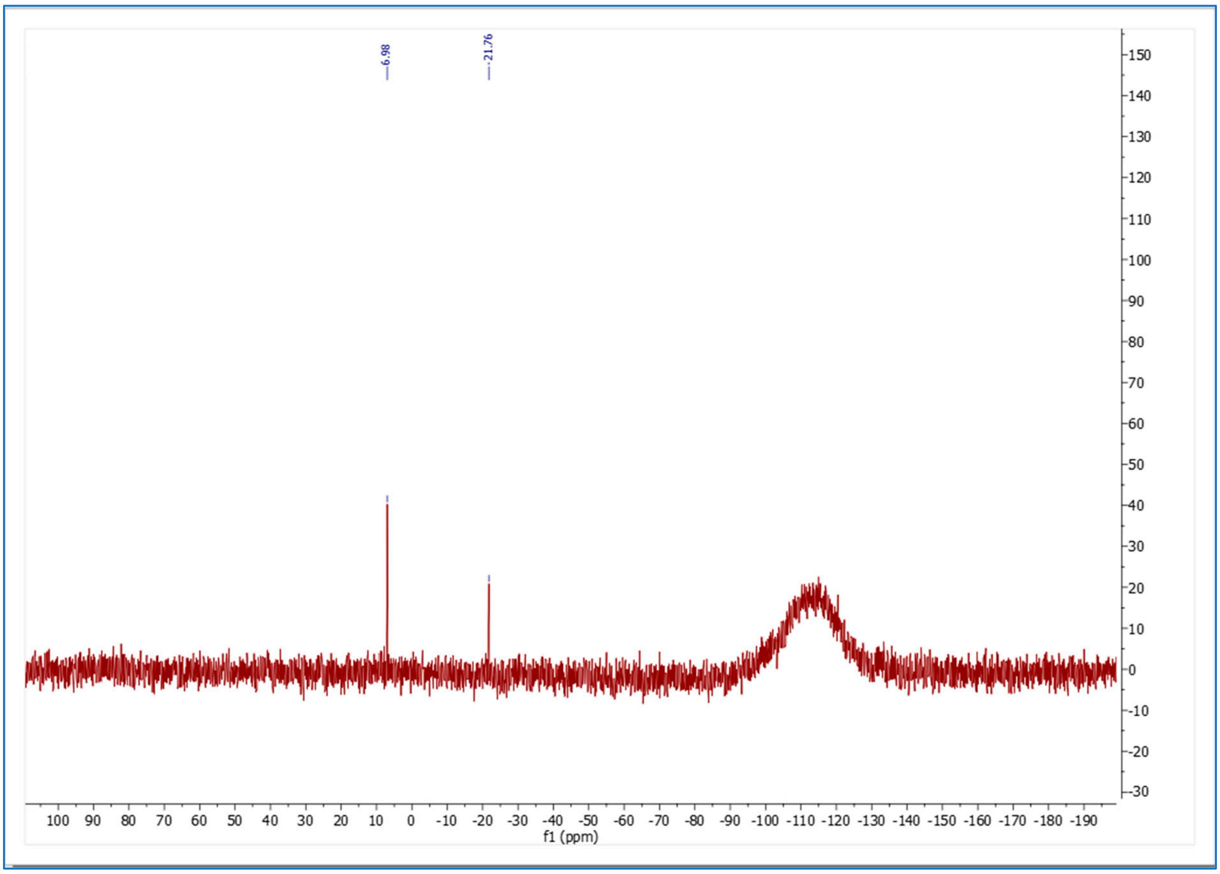

Figure S8. ^{29}Si -NMR spectrum of HOL9.

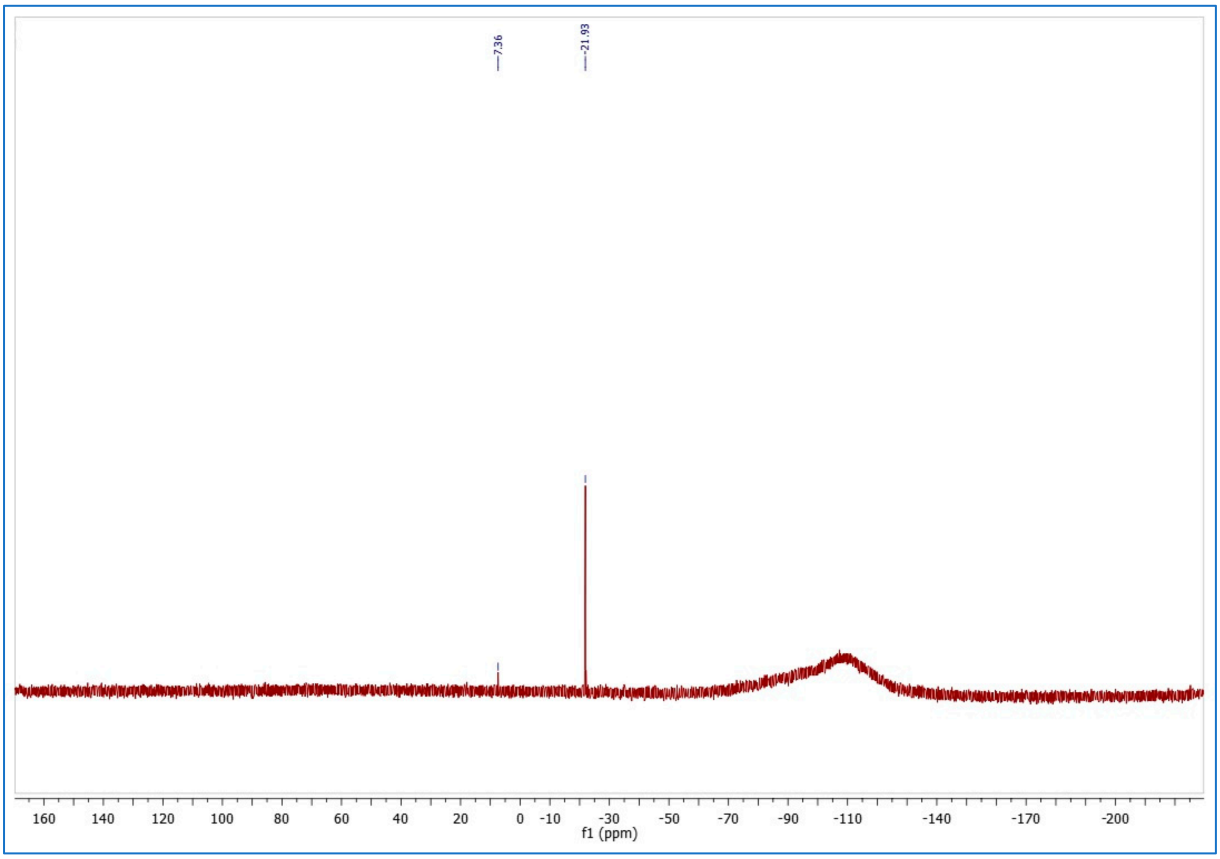

Figure S9. ^{29}Si -NMR spectrum of HOL12.

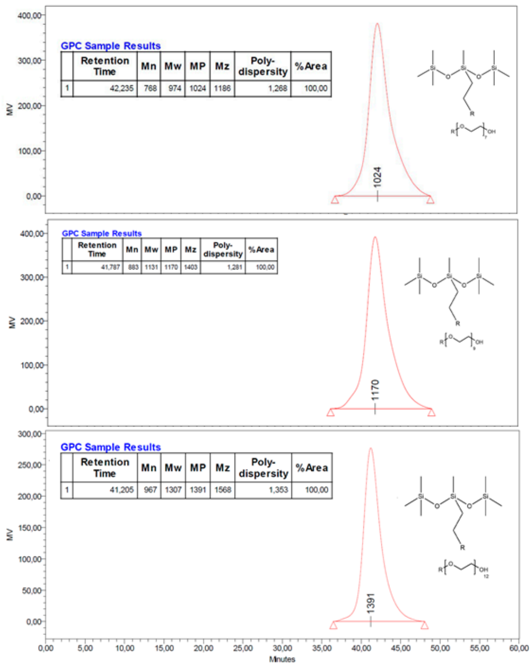

Figure S10. The GPC results of HOL7, HOL9 and HOL12 .

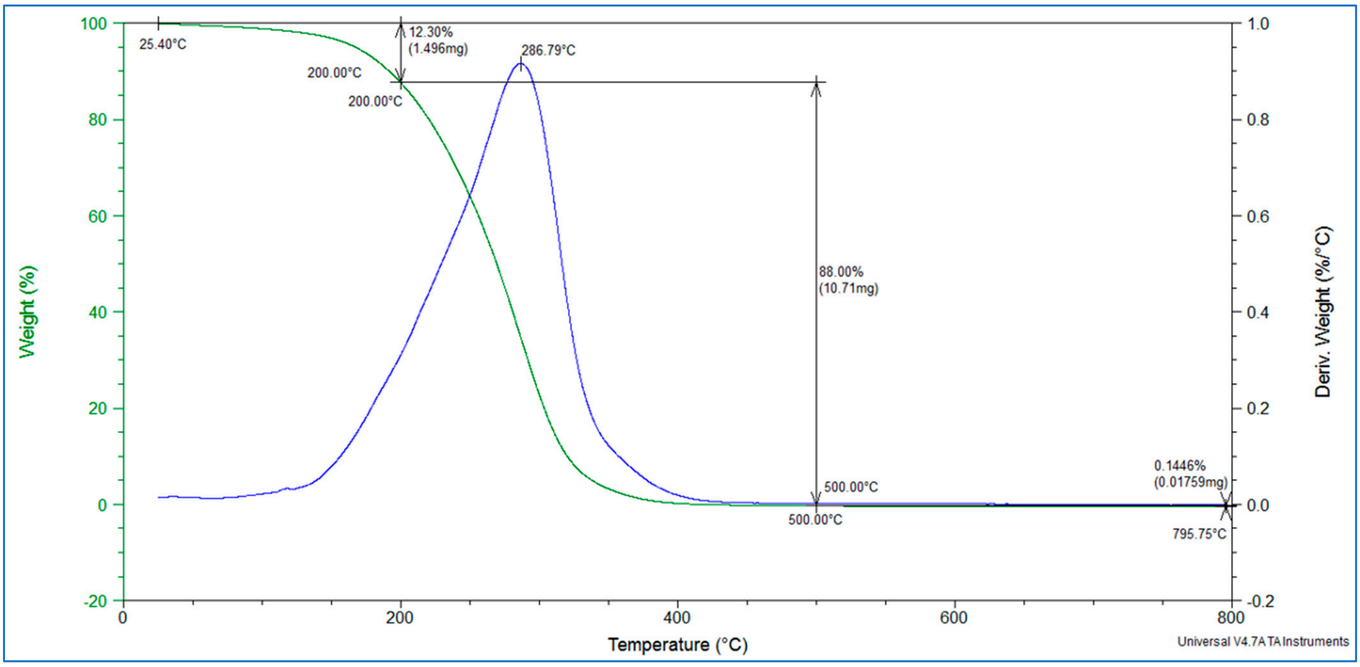

Figure S11. TGA and DTG curves of HOL7.

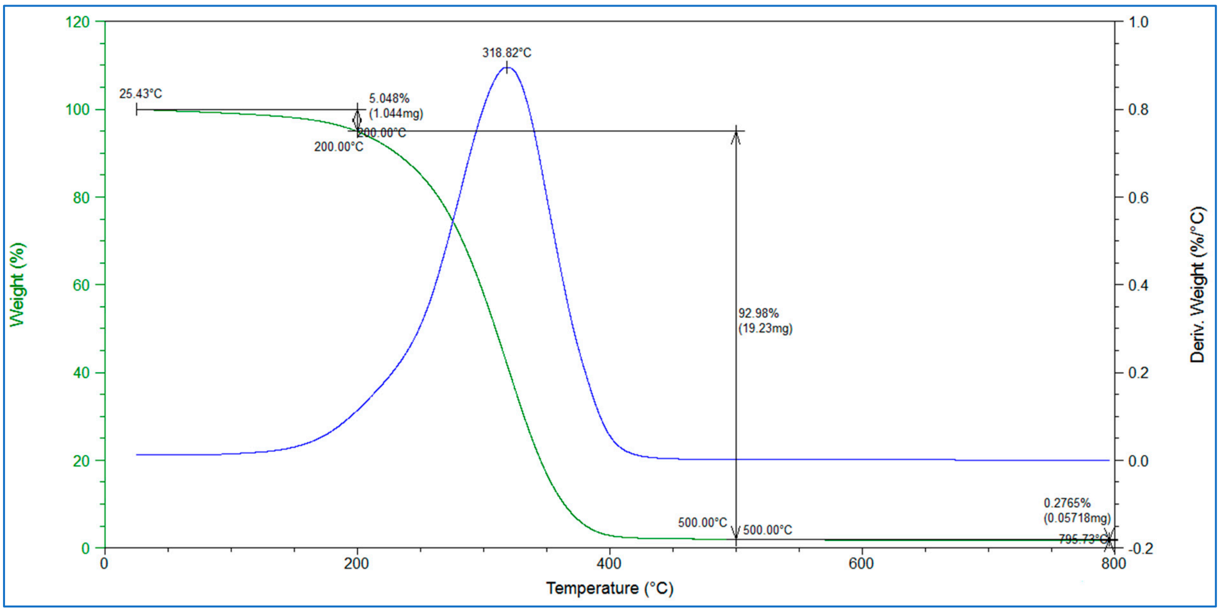

Figure S12. TGA and DTG curves of HOL9.

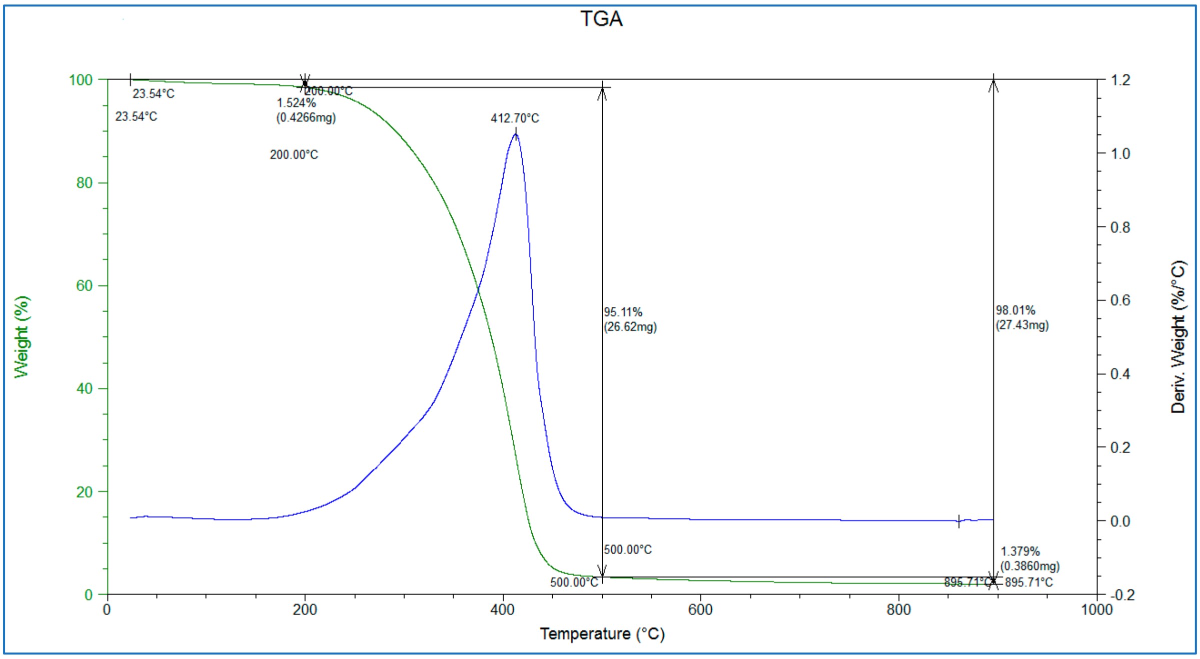

Figure S13. TGA and DTG curves of HOL12.
